# First serological evidence of SARS-CoV-2 natural infection in small ruminants: Brief report

**DOI:** 10.1007/s11259-022-10044-3

**Published:** 2023-01-10

**Authors:** Giovanna Fusco, Lorena Cardillo, Martina Levante, Sergio Brandi, Gerardo Picazio, Michele Napoletano, Alessandra Martucciello, Filomena Fiorito, Esterina De Carlo, Claudio de Martinis

**Affiliations:** 1 Department of Animal Health, Istituto Zooprofilattico Sperimentale del Mezzogiorno, Unit of Virology, Via Salute, 2, 80055 Portici, Naples, Italy; 2 CEINGE Advanced Biotechnologies, Via G. Salvatore, 486, 80131 Naples, Italy; 3 Caserta Section, Istituto Zooprofilattico Sperimentale del Mezzogiorno, 81100 Caserta, Italy; 4 Salerno Section, Istituto Zooprofilattico Sperimentale del Mezzogiorno, 84131 Fuorni, Salerno, Italy; 5 Department of Veterinary Medicine and Animal Production, University of Naples Federico II, 80137 Naples, Italy; 6 Scientific Direction, Istituto Zooprofilattico Sperimentale del Mezzogiorno, 80055 Portici, Naples, Italy

**Keywords:** SARS-CoV-2, Sheep, Goats, Natural infection, Host range, Serology

## Abstract

Severe Acute Respiratory Syndrome Coronavirus 2 (SARS-CoV-2) emerged in late December 2019 and spread worldwide, quickly becoming a pandemic. This zoonotic coronavirus shows a broad host range, including wildlife and domestic animals. Small ruminants are shown to be susceptible to SARS-CoV-2 but, to date, no natural infection has been reported. Herein, we performed a survey for SARS-CoV-2 among sheep and goats in the Campania region of Italy using an indirect multispecies ELISA. Next, positive sera were submitted to virus serum neutralization for the quantification of specific neutralizing antibodies. Out of 612 sheep and goats, 23 were found ELISA positive (3.75%) and 1 of them showed 1:20 neutralizing antibodies titer. No significant difference was found between the two species, as well as between male and female, geographical location and age. Our findings demonstrate that natural infection can occur in flocks in a field situation. Moreover, low susceptibility to SARS-CoV-2 is reported for sheep and goats, nevertheless, the continuous mutations of this virus open new scenarios on viral host range and tropism, highlighting the importance of investigating animal species that could represent ongoing or future possible hosts.

## Introduction

Severe Acute Respiratory Syndrome Coronavirus 2 (SARS-CoV-2), the etiologic agent responsible for Coronavirus Disease 2019 (COVID-19), was first identified in December 2019 in Wuhan, China, and rapidly spread globally, opening the ongoing pandemic scenario. To date, over 545 million confirmed cases of COVID-19 are reported by the World Health Organization (WHO), resulting in more than 6 million deaths worldwide (WHO, 2022a accessed on 5 July 2022). Since the first identification of this novel coronavirus in two dogs in Hong Kong (Sit et al., 2020), several studies have been conducted in order to better characterize the tropism and host range of the virus. These two parameters depend on the ability of the receptor-binding domain (RBD) of SARS-CoV-2 Spike protein (S) to bind the host angiotensin-converting enzyme 2 (ACE2) (Lam et al., 2020; Chen et al., 2020; Eslami et al., 2022). Moreover, it has been proven that the primary structure of ACE2 is highly conserved in vertebrates, thus making several animal species susceptible to SARS-CoV-2 infection (Chen et al., 2020; Damas et al., 2020; Zhai et al., 2020). Indeed, the latest epidemiological data of the World Organization for Animal Health (WOAH) report 676 outbreaks globally, affecting 23 species in 36 countries, involving cats, dogs, minks, otters, pet ferrets, lions, tigers, pumas, snow leopards, gorillas, white-tailed deer, fishing cats, Binturong, South American coati, spotted hyena, Eurasian lynx, Canada lynx, hippopotami, hamsters, mule deer, giant anteaters, West Indian manatees, black-tailed marmosets (WOAH, 2022a). In general, animals show an asymptomatic infection, but when signs develop, they are mainly mild or moderate, nevertheless, some species are particularly susceptible to SARS-CoV-2, as white-tailed deer (Hale et al., 2022) and can experience a severe disease, even fatal such as in minks (Oreshkova et al. 2020; WOAH, 2022b). Thus, the absence or limited clinical signs may lead to underdiagnosed cases of SARS-CoV-2 infection in animal species. Furthermore, it has been demonstrated by experimental and natural infections, that positive animals show low amounts of viral shedding, during a very short time (Murphy and Ly, 2021; Ulrich et al., 2020; Falkenberg et al., 2021; Gaudreault et al. 2021; Cardillo et al., 2022; Fiorito et al., 2022). Therefore, serological examinations, revealing a previous viral infection, are a useful tool to investigate SARS-CoV-2 host range and prevalence in animal species, and, in particular, virus serum neutralization assay (VSN), that is species-independent and is based on the principle of in vitro interaction between specific neutralizing antibodies and the investigated virus (Wernike et al., 2020).

The susceptibility of domestic ruminants to SARS-CoV-2 is currently under investigated, albeit these species are of particular interest since, as for other species, cattle and sheep ACE2 shows affinity to SARS-CoV-2 RBD (Zhai et al., 2020: Di Teodoro et al., 2021) and, in particular, livestock are often in close contact with farmworkers and, during grazing, with potential known SARS-CoV-2 wildlife reservoirs (Gaudreault et al. 2021; Fiorito et al., 2022), such as white-tailed deer in North America (Hale et al., 2022), the recently reported Asian small-clawed and Eurasian otters, (Padilla-Blanco et al., 2022) and/or still unknown susceptible feral species (WOAH, 2022c). Particular concerns have also been highlighted by Lam and colleagues (2020), who, in order to predict infection risks, analyzed a modelling S-protein:ACE2 complex from sheep, by calculating energetic changes of the complex due to species-specific mutations, as well as by examining chemical-physical affinity due to specific groups. The comparison between energetic binding affinity of sheep showed similarity to that from humans. Interestingly, it has been demonstrated that ACE2 orthologs of some small ruminants allow cell entry of SARS-CoV-2, indicating that sheep and goats may harbor as well as spread the virus (Li et al., 2020). Furthermore, an *in silico* study evaluating the interaction between ACE2 receptor and the S protein, has evidenced the susceptibility of sheep (Damas et al., 2020) to SARS-CoV-2 infection and, later, by ex vivo organ cultures (EVOCs) and in vitro experiments, it has been observed that the ovine ACE2-expressing cells of the respiratory tract are able to sustain viral replication (Gaudreault et al. 2021; Di Teodoro et al., 2021). To date, no natural infection in sheep and goats has been observed. Indeed, little information is available for goats and a serological study was performed in Spain in sheep but without revealing anti-SARS-CoV-2 antibodies (Villanueva-Saz et al., 2021). Bosco-Lauth and colleagues (2021) evaluated the susceptibility of livestock to SARS-CoV-2 by experimental infections. They found no infectious virus shedding and no viral RNA was detectable by nasal-oral swabs in sheep, while 2 of 3 goats showed positive results by RT-PCR. Moreover, challenged small ruminants show anti-SARS-CoV-2 detectable antibodies but for a short time after infection and low titers of neutralizing antibodies (Gaudreault et al. 2021; Bosco-Lauth et al. 2021). Altogether, these observations have led to the conclusion that sheep and goats have low susceptibility to SARS-CoV-2 and natural infection is unlikely to occur (Gaudreault et al. 2021; Villanueva-Saz et al., 2021; Bosco-Lauth et al., 2021).

The present study aimed to focus the attention to these neglected ruminant species in order to evaluate whether natural infection in flocks and the development of neutralizing antibodies in a natural environment could occur together with their epidemiological role.

## Materials and methods

### Sample collection and data management

From January to April 2022, resulting in a time span of intense SARS-CoV-2 circulation in Campania region, a total of 612 serum samples were obtained by 488 sheep and 124 goats, belonging to 24 different farms located in two provinces of Campania region, Italy. Animals were divided, on the basis of the age, in three classes, 0–5 years, 5–10 years and over 10 years. All the sera were sampled by the Local Veterinary Health Authorities for the national eradication plan for ovine brucellosis. Blood was collected from the jugular vein using sterile evacuated tubes with Serum Clot Activator (Becton, Dickinson and Company, Plymouth, UK) following the dictates of the relevant guidelines and regulations on animal welfare. Samples were transported to the Istituto Zooprofilattico Sperimentale del Mezzogiorno laboratories under refrigeration conditions and after centrifugation at 1300 X g for 10 min (Centrifuge 5810, Eppendorf, Hamburg, Germany), aliquots of the sera were frozen at − 20 °C until use.

For this study, ethical review and approval were waived because of its retrospective nature and sample collection was conducted during official routine activities, thus in accordance to national and regional regulation and internal policy, additional ethical approval was deemed unnecessary.

### Enzyme-linked immunosorbent assay (ELISA) antibody test and virus serum neutralization

All the available sera collected in the aforementioned time span underwent serological examination by indirect Enzyme-Linked Immunosorbent Assay (ELISA), carried out in Biosafety Level 2 (BSL-2) laboratory in order to detect the presence of IgG anti-SARS-CoV-2 nucleocapsid (N) on serum samples, using the ID Screen SARS-CoV-2 Double Antigen Multi-Species ELISA Kit (ID VET, Montpellier, France) as per the manufacturer’s instructions. Accordingly, 25 µl of Dilution Buffer were dispensed in the plate and 25 µl of serum of each animal were added, in the presence of the Negative and Positive Controls. After an incubation of 45 min at 37 °C, the wells were discharged and washed 5 times using 300 µl of Wash Solution. Next, 100 µl of diluted purified N protein recombinant antigen horseradish peroxidase conjugate, with a 1X final concentration, were added, incubated for 30 min at 21 °C, discharged and washed 5 times as aforementioned. Finally, after the addition of 100 µl of Substrate Solution, with an incubation of 20 min at 21 °C in the dark, 100 µl of Stop Solution were added to stop the reaction. The microplate was read using iMark Microplate Absorbance reader (Bio-rad Laboratories, Hercules, California, USA) at 450 nm. The test was considered valid if the Positive Control OD value was > 0.350 and the ratio of Positive/Negative Control was > 3. Thus, following the datasheet guidelines, samples showing S/P% < 50% were considered negative, S/P% between 50 and 60% were considered doubtful, while samples with S/P% > 60% were considered positive.

ELISA tested doubtful and positive samples were next submitted to Virus Serum Neutralization (VSN) test in BLS-3 laboratory with a standardized micro-method already described (Cardillo et al., 2022). Briefly, sera were first heat-inactivated at 56 °C for 30 min and 10-fold pre-dilution was performed in sterile 96-well microplates, followed by 2-fold serial dilution up to 1:10,240. SARS-CoV-2 hCoV-19/Italy/CAM-INMI-32803-66/2020 strain (EPI_ISL_493333), previously titrated according to the Spearman–Kärber method (Kärber, 1931) on VeroE6 cells, was added using 100 TCID50/50 µL titer (corresponding to 2000 TCID50/mL) and incubated at 37 °C ± 1 °C for 1 h in microaerophilic atmosphere. Vero E6 cell suspension of approximately 1.5 × 10^5^ cells/mL was added at a concentration to reach monolayer confluence in 24 h and incubated at 37 °C ± 1 °C for up to 72 h. The plate was first read at 24 h to evaluate the cytotoxic effect and the final readings were performed at 48 and 72 h for the cytopathic effect, by using the inverted microscope (Axioscope 5, Carl Zeiss, Oberkochen, Germany). Contextually, a second plate was prepared containing reference negative and positive sera, virus and cell controls. The absence of cytopathic effect in VeroE6 cells at 72 h post-inoculation was used to determine the presence of neutralizing antibodies and the titer was established on the basis of the highest dilution of serum that prevented infectivity. The samples were considered as positive when a titer ≥ 1/20 was observed and as negative with a titer < 1/20.

### Statistical analysis

Prevalence was calculated at 95% confidence level according to the Wilson method. True Prevalence (TP) was calculated according to Sensitivity (Se) and Specificity (Sp) reported in the datasheet of the internal validation protocol of the ID Screen SARS-CoV-2 Double Antigen Multi-Species ELISA Kit. All statistical analyses were performed by using EpiTools online software, available online at https://epitools.ausvet.com.au/. Results were considered statistically significant with a p value < 0.05.

## Results

A total of 612 sera, 488 sheep and 124 goats, belonging to 24 different farms, in particular, 6 of them located in Caserta and 18 in Salerno province, were tested for SARS-CoV-2 by using IgG anti-SARS-CoV-2 nucleocapsid (N) ELISA. All the farms had an officially free of brucellosis status.

We accounted for Sensitivity (Se) (100% for both species) and Specificity (Sp) (98.9% for sheep and 98.8% for goats) from the ELISA test validation protocol (Laidoudi et al., 2021) to assess true prevalence (TP). Thus, out of 612 small ruminants, 23 were found ELISA positive, with an overall Apparent Prevalence (AP) of 3.75% (2.52–5.58 95% CI) and 2.69% TP (1.4–4.56 95% CI). Next, it was evaluated whether geographic area, species, sex and age could play a role in the seroprevalence. Results showed that no significant difference was found between Salerno (AP 3.74%; TP 2.67%) and Caserta (AP 3.84%; TP 2.78%) provinces (p = 0.7012), as well as no difference was revealed between sheep and goat seropositive cases (p = 0.04806), male and female (p = 0.5345) or age classes (p = 0.4232) (Table 1).

**Table 1:** Seroprevalence of SARS-CoV-2 in small ruminants and statistical evaluation of geographic area, species, sex and age factors

	Total	ELISA Positive					
		N	AP %	95% CI	TP %	95% CI	P value
**Province**							
Salerno	508	19	3.74	2.41–5.77	2.67	1.23–4.73	0.7012
Caserta	104	4	3.84	1.51–9.47	2.78	0.23–8.47	
**Species**							
Sheep	488	17	3.48	2.19–5.51	2.41	1.02–4.45	0.4806
Goats	124	6	4.83	2.24–10.16	3.78	1.04–9.35	
**Sex**							
Female	577	21	3.64	2.39–5.5	2.57	1.22–4.47	0.5345
Male	35	2	5.71	1.58–18.61	4.67	0–18.64	
**Age**							
0–5 y	401	18	4.48	2.86–6.98	3.43	1.75–6.03	0.4232
5–10 y	167	4	2.39	0.94–6	1.31	0–5.12	
> 10 y	44	1	2.27	0.4–11.18	1.19	0–11.12	
**Total**	**612**	**23**	**3.75**	**2.52–5.58**	**2.69**	**1.4–4.56**	

**AP**: Apparent prevalence; **TP**: True prevalence; **95% CI**: 95% Confidence Interval. Results were considered statistically significant with a p value < 0.05

It was also investigated SARS-CoV-2 herd prevalence and it was found that, out of 24 farms, 13 (AP 54.16%; TP 53.66%) showed at least one positive animal, in particular, out of 6 farms in Caserta and 18 in Salerno province, 3 (50%) and 10 (55.5%), respectively, were found to have a positive animal, showing an intra-herd prevalence ranging from 0 to 6.45% (mean value 2.24%) in Caserta and 0–33.33% in Salerno (mean value 6.76%) (Table 2). Farm owners and the animal caretakers were asked whether in the time span of the study they were found SARS-CoV-2 positive. Results showed that, out of 13 positive herds, 5 (38.4%) have had a human case of COVID-19, in particular, one in Caserta province (1/3; 33.3%) and had a human case of COVID-19 and four in Salerno (4/10; 40%).

**Table 2:** Distribution of ELISA tested positive animals and intra-farm prevalence

		ELISA		
Province	Total animal/farm (n)	Positive (n)	Prevalence (%)	
**Caserta**	4	0	0	
	7	0	0	
	35	1	2.85	
	31	2	6.45	
	24	1	4.16	
	3	0	0	
**Salerno**	18	0	0	
	99	4	4.04	
	41	0	0	
	8	1	12.5	
	5	1	20	
	14	0	0	
	19	2	10.52	
	9	0	0	
	4	1	25	
	2	0	0	
	3	1	33.33	
	9	1	11.11	
	11	1	9.09	
	112	1	0.89	
	3	0	0	
	5	0	0	
	6	0	0	
	140	6	4.28	
**Total**	**612**	**23**	**3.75**	

Seropositive samples were then submitted to virus serum neutralization assay, resulting in the detection of specific neutralizing antibodies in a sheep with 1:20 titre belonging to a farm from Caserta province, which had no human report of COVID-19.

## Discussion

The error-prone nature of RNA polymerase RNA dependent of SARS-CoV-2 has caused a rapid occurrence of several mutations, leading to the emergence of new variants, some of which are classified as “Variants of Concern” (VOCs). These VOCs are characterized by an enhanced pathogenicity, morbidity or immune-escape to previous infection and/or vaccination (Yi et al., 2021; CDC, 2022). Furthermore, some variants have been suggested to show an expanded host tropism, becoming capable of infecting new species (Pickering et al., 2022). The first reports of SARS-CoV-2 in domestic and wild animals have led international organizations to recommend researchers, following a holistic approach, to focus their efforts on the study and understanding of virus host range, transmissibility and the role of animals in the epidemiology of this novel coronavirus both in farmed and companion animals (WOAH, 2022d; FAO, 2021; WHO, 2022b). Although it is described that the preferred techniques to evaluate SARS-CoV-2 exposure in animals should be the association of antibodies or antigen detection, molecular biology etc., basically, the use of broad serological investigations represents the main tool to detect positive animals than the only use of virological examination, due to a limited time span of viral shedding, as reported by WOAH (WOAH, 2022d).

In general, SARS-CoV-2 infection in animals is mainly considered an anthropozoonoses, nevertheless, the acquisition of unique animal-linked mutations has probably induced the emergence of novel mutants responsible for spillback events to humans, as demonstrated by farmed minks and, recently, hamsters, white-tailed deer and a cat (Pickering et al., 2022; Hammer et al., 2021; Yen et al., 2022). Moreover, the latest Omicron VOC, that shows high divergence from any reported SARS-CoV-2 previous variant, has been hypothesized to evolve in mice by a spillover occurred from humans to mice and, by acquiring numerous mutations, jumped back to humans (Wei et al., 2021). Therefore, the continuous mutation process of this virus underlines the importance of investigating on several other animal species that, although are currently considered to have low susceptibility to the infection, could represent future possible hosts.

In these last two years, most efforts have been directed to evaluate SARS-CoV-2 infection in companion animals, minks and white-tailed deer, that have shown high susceptibility to this virus, nevertheless, considerable gaps remain regarding ruminants, particularly sheep and goats, that are conventionally housed in large groups and are in close contact with farmworkers during farming processes (Gaudreault et al. 2021; Di Teodoro et al., 2021). To date, no study reports natural infection in these species by both serological and biomolecular examinations (Villanueva-Saz et al., 2021; Cerino et al., 2021). The available information about small ruminants comes from experimental infections, where it has been demonstrated that sheep show low susceptibility to SARS-CoV-2 infection while higher susceptibility has been revealed in goats. Indeed, challenged sheep show mainly a subclinical course upon exposure, no RNA detection by RT-PCR or low amounts of virus shedding, and it has been demonstrated that sheep are able to transmit the virus to sentinels (Gaudreault et al. 2021; Bosco-Lauth et al., 2021). Nevertheless, natural infection in this species is reported to unlikely occur and the risk of transmission to co-mingled is low (Gaudreault et al. 2021; Bosco-Lauth et al., 2021). Goats, on the other hand, are able to share the virus by nasal and/or oral route, suggesting that these animals may be minimally permissive to the infection (Bosco-Lauth et al., 2021).

The present study demonstrates that sheep and goats can be naturally infected by SARS-CoV-2, even with poor immune response, showing a variable range of intra-herd prevalence.

Albeit by experimental infection it can be deduced that goats are more susceptible than sheep (Bosco-Lauth et al., 2021), no statistical difference between these two species was observed (p = 0.4806), as well as for age, sex and geographic area of the farms. Furthermore, our field results are in accordance with the findings of the two study groups of Bosco-Lauth (2021) and Gaudreault (2021) that observed by serological examinations, that small ruminants show low titre of neutralizing antibodies. Indeed, Gaudreault (2021) observed that, among 10 infected sheep, all the animals showed detectable antibodies by ELISA but only one of them developed neutralizing antibodies with 1:20 titre. Moreover, as it has been described that most of livestock show a rapid decay of neutralizing antibodies (Bosco-Lauth et al., 2021), the identification of a sheep with specific neutralizing antibodies in our study might be suggestive of a recent natural infection. Therefore, the poor and short time of SARS-CoV-2 shedding after exposure, together with the absence of a robust and long-term persistence of neutralizing antibodies that characterizes these two animal species (Gaudreault et al. 2021; Bosco-Lauth et al., 2021) could be at the basis of the lack of identification of positive animals during livestock investigations (Villanueva-Saz et al., 2021; Cerino et al., 2021). Alternatively, it can be also hypothesized that the absence of confirmation to VSN of the ELISA tested positive animals and the low titre of the only confirmed case could be ascribed to possible non-specific inhibitors in the sera of sheep and goats, that could interfere with the neutralization test (Jouneau et al., 2020), even when heat inactivation prior to the test is performed.

Nevertheless, serological examinations, represent a good screening tool to investigate SARS-CoV-2 exposure in several animal species, although they do not represent the main hosts (Wernike et al., 2021) and it is debated that there might be cross-reactions with other betacoronaviruses (Hicks et al., 2021; Laidoudi et al., 2021), but, when associated to species-independent confirmatory tests, such as VSN, reliable results can be obtained. Moreover, other studies on SARS-CoV-2 multi-species ELISA tests showed no cross-reaction between SARS-CoV-2 and Bovine coronavirus antibodies, albeit based on the RBD domain (Wernike et al., 2021). Thus, in order to minimize the bias of the seropevalence, the true prevalence was calculated, considering the Sensitivity and Specificity of the ELISA test.

The epidemiological considerations regarding our findings are difficult to interpret. In particular, inter-species transmission from humans to animals or intra-species within the flock are not easy to explore. It can be supposed that in areas with large population of small ruminants, associated with the presence of SARS-CoV-2 infection in humans, close contact between livestock and farmworkers may have caused a reverse zoonotic infection as hypothesized for other highly sensitive animal species, such as minks, cats and dogs and for lower susceptible species, as cattle (Fiorito et al., 2022). Albeit, our results showed that only the 38.4% of the positive herds referred a case of human COVID-19, thus opening the question about further sources of infection for these animals, probably through the environment.

Despite the ACE2 distribution in the respiratory tract, poor in vivo susceptibility of sheep has been described, suggesting that this species is unlikely to be effective intermediate host for coronavirus evolution (Ulrich et al., 2020; Gaudreault et al. 2021; Di Teodoro et al., 2021; Vergara-Alert et al., 2021). Nevertheless, seroprevalence studies in certain species such as small ruminants, can be an important data deduction tool for monitoring the environment circulation of the pathogen. The source and timing of infection in livestock, as well as their potential spread of the virus, is not always clear and deducible. However, these actions are necessary to focus on the containment of the pathogen through wide surveillance and control programs.

## Conclusion

The numerous mutations of SARS-CoV-2 over time have led to the emergence of new lineages and the discovery of new animal species susceptible to viral infection and, in some cases, also to spillback events. This underlines the importance of surveillance in animals for SARS-CoV-2 in order to have high level of preparedness to adverse events, such as spillbacks from further animal species, especially those that live in close contact with humans and in gathering, that could represent an ongoing or future source of infection for highly susceptible species.

## Data Availability

Not applicable. All data supporting the findings of this study are included within the manuscript.
